# Preparation and properties of starch-based carbon aerogel/FeCoNi composite wave-absorbing materials

**DOI:** 10.1039/d6ra00299d

**Published:** 2026-03-19

**Authors:** Yue Wang, Xiang Zhou, Hu Gu, Sen Yang, Xiaoyun Long, Shuang Zhai, Qilong Sun

**Affiliations:** a College of Textiles and Clothing, Nantong University Nantong 226019 China zhai_shuang@163.com sunqilong001@ntu.edu.cn; b Jiangsu College of Engineering and Technology Nantong 226007 China; c Hangzhou Institute of Quality & Metrology Hangzhou 310019 China; d Jiangsu Key Laboratory of Market Supervision (Research on Safety Performance Evaluation of Personal Protective Equipment) Taizhou 225300 China; e National & Local Joint Engineering Research Center of Technical Fiber Composites for Safety and Protection, Nantong University Nantong 226019 China

## Abstract

To address the increasingly serious electromagnetic pollution problem in the era of 6G communication, electromagnetic wave-absorbing materials have gradually become a research focus in modern society. In this work, cassava starch was selected as the precursor, and FeCoNi precipitates were introduced. Starch-based aerogel/FeCoNi composite wave-absorbing materials (FeCoNi/SA) were obtained through sol–gel and freeze-drying processes. Afterward, starch-based carbon aerogel/FeCoNi composite wave-absorbing materials (FeCoNi/SCA) were produced *via* high-temperature carbonization in a vacuum muffle furnace. The microstructure, chemical features, and wave-absorbing performance of FeCoNi/SCA were examined, and the absorption mechanism was discussed. The results indicated that at a matching thickness of 3 mm, the minimum reflection loss (RL_min_) reached −60.89 dB, and the effective absorption bandwidth (EAB) was 6.27 GHz (7.31–13.58 GHz). When the matching thickness increased to 3.5 mm, the EAB expanded to 7.91 GHz (5.61–13.52 GHz). This type of material combines a lightweight porous architecture and a magnetic–dielectric synergistic loss effect, providing a feasible strategy for the development of high-efficiency and environmentally friendly wave-absorbing materials.

## Abstract 2

FeCoNi-doped starch carbon aerogels achieve superior microwave absorption (RL_min_ = −60.89 dB, EAB = 7.91 GHz) *via* magnetic–dielectric synergy, offering a green strategy for efficient electromagnetic attenuation.

## Graphical abstract

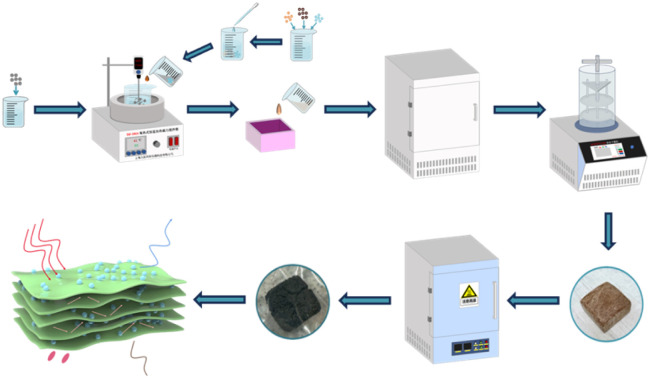

## 1 Introduction

With the rapid development of modern electronic technology, especially the gradual application of sixth-generation mobile communication (6G) technology, electromagnetic waves have brought convenience to society, but also caused increasingly serious problems of electromagnetic interference and electromagnetic radiation pollution. Electromagnetic radiation not only interferes with the normal operation of precision electronic equipment, but also poses a potential threat to human health, and has been listed as the fourth largest environmental pollution source after water pollution, air pollution, and noise pollution.^1^ Therefore, the development of electromagnetic wave absorption materials with high-efficiency absorption, broad bandwidth, light weight, and ultra-thin thickness to achieve effective protection and attenuation of electromagnetic waves has become a research hotspot to meet the practical needs of many fields.

Electromagnetic wave absorption materials are a class of functional materials that can absorb and attenuate electromagnetic waves. Their core function is to convert incident electromagnetic wave energy into heat or other forms of energy for dissipation. Ideal microwave absorption materials should simultaneously meet the requirements of “strong absorption, broad bandwidth, thin thickness, and light weight” to adapt to the complex application environments in modern military stealth, civil electronic compatibility, aerospace, and other fields.^2^ For example, the wave-absorbing coatings on the surface of military aircraft need to be both high-temperature resistant and lightweight to cope with radar detection, while the wave-absorbing materials for portable electronic devices need to be ultra-thin and flexible to suppress electromagnetic interference. In recent years, researchers have achieved broadband sound absorption through metamaterial structure design^3,4^ and optimized electromagnetic parameters *via* high-entropy strategies.^5^ These advanced design concepts provide new ideas for the development of high-performance microwave absorption materials. However, traditional microwave absorption materials (such as ferrites) often suffer from high density and narrow absorption bandwidth, making it difficult to meet the development demands of multi-functional integration.^6^

Aerogel materials exhibit unique advantages in the field of microwave absorption due to their ultra-high porosity, extremely low density, and tunable electrical conductivity. The porous structure of aerogels facilitates the multiple reflection and scattering of electromagnetic waves, thereby enhancing energy dissipation.^7^ At present, various aerogel wave-absorbing materials have been developed, such as SiC@SiO_2_ nanofiber aerogel (SiC@SiO_2_ NFA), cellulose-chitosan/polyaniline hybrid aerogel (CCPA), and TiN-decorated SiOC ceramic fiber composite (SiOC@TiN), and certain wave-absorbing properties have been obtained.^8–10^ Nevertheless, the preparation processes of these materials usually involve complicated procedures or expensive raw materials, limiting their large-scale applications. Therefore, it is of great significance to explore green, low-cost, and high-performance aerogel wave-absorbing materials.

As a natural polysaccharide polymer, starch has the advantages of wide sources, renewability, and good biocompatibility. Different from poorly soluble polysaccharides such as cellulose and chitosan, starch can form a stable hydrogel network through a simple sol–gel process without adding cross-linking agents, making it an ideal precursor for the preparation of biomass-derived carbon aerogels. The carbonized starch-based carbon aerogel not only retains a lightweight and porous structure, but also possesses certain electrical conductivity, which can generate dielectric loss. In addition, by introducing magnetic metal particles such as FeCoNi, a magnetic–dielectric synergistic loss effect can be constructed to further improve the microwave absorption performance. The green and environmentally friendly characteristics of starch-based materials can also meet the practical requirements of low pollution in civil electronics and other fields.

Based on the above background, in this study, starch-based aerogel was prepared using cassava starch as the precursor *via* sol–gel and freeze-drying processes, and then FeCoNi-doped starch-based carbon aerogel (FeCoNi/SCA) was obtained through high-temperature carbonization in a vacuum muffle furnace. The phase composition, micromorphology, element distribution, and electromagnetic parameters of the material were systematically investigated. The microwave absorption performance in a broadband range was emphatically analyzed, and the intrinsic relationship between the wave absorption properties and the structure was revealed. This work aims to provide a new design strategy and experimental basis for the development of green, lightweight, and high-efficiency biomass-derived microwave absorption materials.

## 2 Experiments

### 2.1 Raw materials and reagents

Cassava starch used in this study was purchased from Xinxiang Liangrun Whole Grain Co., Ltd FeCl_3_, CoSO_4_·7H_2_O, and NiSO_4_·6H_2_O were obtained from Shanghai Macklin Biochemical Technology Co., Ltd (purity: AR grade). NH_3_·H_2_O was purchased from Xilong Scientific Co., Ltd (purity: 99.7%). Deionized water used in the experiment was prepared in the laboratory.

### 2.2 Equipment and instruments used in the experiment

BSA224S analytical balance was produced by Sartorius Scientific Instruments (Beijing) Co., Ltd; DF-101S constant-temperature magnetic stirrer with heat-collecting function was supplied by Gongyi Yuhua Instrument Co., Ltd; LC-OES-60 cantilever electric stirrer was provided by Shanghai Lichengbangxi Instrument Co., Ltd; SCIENTZ-12N freeze dryer was manufactured by Ningbo Xinzhi Biotechnology Co., Ltd; SY-10 compressed air freezer dryer was made by Hebei Bangyi Machinery Technology Co., Ltd; BNT-SP103 nitrogen generator was produced by Baonite (Hebei) Energy-Saving Technology Co., Ltd; and the ZSX-15-14 vacuum muffle furnace was manufactured by Xinite (Beijing) Technology Co., Ltd

### 2.3 Experimental methods

**Fig. 1:**
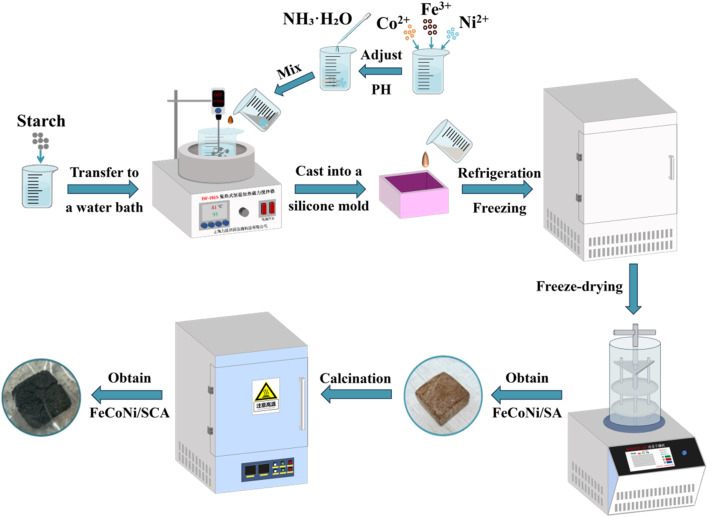
Schematic diagram of the preparation process for FeCoNi/SCA.

The preparation process of FeCoNi/SCA is shown in Fig. 1. Starch was gelatinized at high temperature, doped with FeCoNi precipitates, and then allowed to stand and retrograde to form hydrogels. The water in the gel was frozen into a solid state through low-temperature freezing, and the frozen gel was converted into an aerogel using a vacuum freeze dryer. Finally, the aerogel was transformed into a carbon aerogel through high-temperature calcination in a muffle furnace.

#### 2.3.1 Preparation of FeCoNi/SA

A certain amount of FeCl_3_, CoSO_4_·7H_2_O, and NiSO_4_·6H_2_O was weighed and mixed in a beaker at a ratio of 4 : 1 : 1. Then, 20 mL of NH_3_·H_2_O was added, followed by rapid stirring to fully disperse them in the solution, producing a reddish-brown viscous liquid. Ammonia water was slowly added while stirring until the pH reached 7, and stirring was continued for some time to obtain FeCoNi precipitates. The total molar concentration of FeCl_3_, CoSO_4_·7H_2_O, and NiSO_4_·6H_2_O was 0.6 mol·L^−1^.

A certain amount of cassava starch was weighed and poured into a beaker containing 140 mL of deionized water, followed by rapid stirring (starch mass fraction: 10%) to disperse the starch uniformly and form a starch milk solution. It was then placed in a 90 °C water bath, and a cantilever electric stirrer was used for sufficient stirring and gelatinization at 600 rpm for a set time. The prepared FeCoNi precipitates were added during gelatinization, and gelatinization was continued for 30 min. Afterward, the starch paste was poured into a silicone mold and covered with plastic wrap to prevent water evaporation.

After cooling the starch paste to room temperature, it was transferred to a 4 °C refrigerator for standing and retrogradation for 24 h to obtain FeCoNi/starch-based hydrogel (FeCoNi/SH). It was then placed in a −18 °C freezer for 8 h, and further pre-frozen in a −80 °C freezer for 5 h. Finally, freeze drying was carried out for 48 h to obtain FeCoNi/SA. When the FeCoNi doping was 0 (*i.e.*, cassava starch aerogel), the sample was labeled SA.

#### 2.3.2 Preparation of FeCoNi/SCA

Under nitrogen protection, FeCoNi/SA was calcined in a vacuum muffle furnace at 750 °C for 2 h, and then cooled to room temperature in a nitrogen atmosphere (heating rate: 5 °C min^−1^) to obtain FeCoNi/SCA. When the FeCoNi doping was 0 (*i.e.*, cassava starch carbon aerogel), the sample was labeled SCA. All aerogels had the same starch content during preparation and were produced using the same mold and method.

### 2.4 Testing and characterization

#### 2.4.1 Electromagnetic parameters

An AV3672C vector network analyzer (manufactured by the 41st Research Institute of China Electronics Technology Group Corporation) was used to measure electromagnetic parameters of the samples in the frequency range of 2–18 GHz. Sample preparation was as follows: FeCoNi/SCA was ground into powder and uniformly mixed with paraffin at a mass ratio of 1 : 4, then pressed into coaxial rings with an inner diameter of 3.00 mm and an outer diameter of 7.00 mm.

#### 2.4.2 Raman spectroscopy

A micro-Raman spectrometer (Raman, Horiba LabRAM, Japan) was used to characterize the degree of graphitization of the material, and structural information of carbon was analyzed using Raman spectroscopy. The excitation wavelength was 532 nm, and the spectral range was 50–4000 cm^−1^. The sample was ground into powder for testing.

#### 2.4.3 Magnetic characterization

A vibrating sample magnetometer (VSM, LakeShore, USA) was used to characterize static magnetic properties of the material. The instrument's magnetic moment measurement sensitivity at room temperature was 5× 10^−7^ emu, and the maximum applicable magnetic field was 2.17 T (with a pole spacing of 16.2 mm). The sample was ground into powder for testing.

#### 2.4.4 Phase composition

An X-ray diffractometer (XRD, Rigaku MiniFlex 600, Japan) was used to analyze the phase composition of FeCoNi/SCA samples. The system used Cu Kα radiation with a tube voltage of 40 kV and a tube current of 15 mA. The diffraction angle (2*θ*) ranged from 5° to 95°, with a step size of 0.02°.

#### 2.4.5 Surface morphology

A field-emission scanning electron microscope (SEM, Gemini SEM 300, Carl Zeiss, Germany) was used to observe the cross-sectional microstructure of SCA and FeCoNi/SCA samples. To minimize charge accumulation, a thin layer of gold was sputtered on the sample surface before characterization.

#### 2.4.6 Element distribution and chemical composition

X-ray photoelectron spectroscopy (XPS, Thermo Fisher K-Alpha) was used to analyze the elemental distribution and chemical composition of FeCoNi/SCA samples. The test employed a monochromatic Al Kα X-ray source with an energy of 1486.6 eV and a power of 150 W.

## 3 Results and discussion

### 3.1 Characterization of wave-absorbing properties

Reflection loss (RL) and effective absorption bandwidth (EAB) are important indicators for evaluating the wave-absorbing performance of materials. Based on transmission line theory, the input impedance (*Z*_in_) and reflection loss (RL) of the samples were calculated according to eqn (1) and (2):^11^

1 *Z*_in_ = *Z*_0_(*µ*_r_/*ε*_r_)^1/2^ tan *h*[*j*(2π*fd*/*c*)(*µ*_r_*ε*_r_)^1/2^]

2
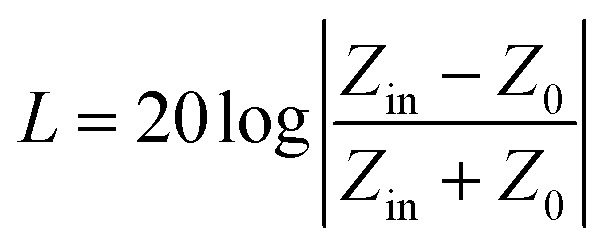
where *Z*_0_ is the impedance of free space (377 Ω), *ε*_r_ and *µ*_r_ are the complex permittivity and complex permeability of the material; *c* is the speed of light; *d* is the matching thickness; *f* is the frequency; tanh is the hyperbolic tangent function; *j* is the imaginary unit. These equations indicate that the microwave absorption performance is strongly dependent on the dielectric and magnetic properties of the material.

RL is generally negative; the smaller its value, the stronger the wave-absorbing ability of the material. When RL is less than −10 dB, it indicates that more than 90% of electromagnetic waves are absorbed. RL lower than −10 dB is commonly used as the benchmark for evaluating wave-absorbing capacity. The electromagnetic wave frequency band with RL lower than −10 dB is called the effective absorption frequency band, and its frequency width range is defined as the EAB.^12^

**Fig. 2:**
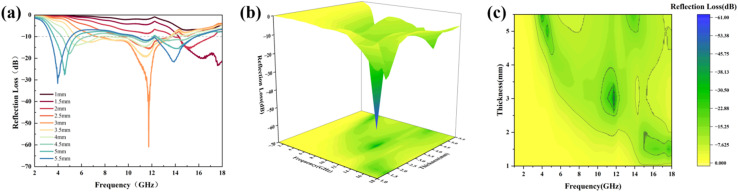
(a) Reflection loss of FeCoNi/SCA at different thicknesses, (b) 3D plot of reflection loss values, (c) 2D contour plot.

Fig. 2 shows the electromagnetic wave absorption of FeCoNi/SCA with different thicknesses in the range of 2–18 GHz. It can be seen that FeCoNi/SCA exhibits good wave-absorbing performance, with an effective absorption band when *d* is 1.5–5.5 mm. At a matching thickness of 3 mm, RL_min_ reaches −60.89 dB (11.73 GHz), and the EAB is 6.27 GHz (7.31–13.58 GHz). When the matching thickness is 3.5 mm, the EAB expands to 7.91 GHz (5.61–13.52 GHz).

Fig. 2(c) is the 2D contour diagram obtained from the test. Overall, as the sample thickness increases, the absorption peak gradually shifts toward the low-frequency direction. This is consistent with the quarter-wavelength cancellation model:^13^3
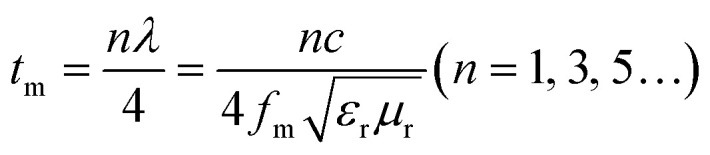
where *t*_m_ represents the matching thickness of the composite material, *λ* represents the wavelength of electromagnetic waves, and *f*_m_ represents the matching frequency. There is an inverse relationship between the peak frequency and the absorber thickness. From eqn (3), it can be understood that when the material thickness is designed to be an odd multiple of a quarter of the incident wavelength, an optimal interference effect is achieved. In this case, the phase difference between the incident electromagnetic wave and the reflected wave is half a cycle, so the amplitudes of electromagnetic waves in the two directions can cancel each other. Therefore, this study also suggests that, besides dielectric loss and magnetic loss, interference cancellation plays an important role in attenuation.

It can be observed that the FeCoNi/SCA prepared in this study exhibits excellent wave-absorbing properties and clear multi-peak loss characteristics. “Multi-peak loss” refers to the presence of minimum reflection loss values <−10 dB in multiple (≥2) frequency bands. The occurrence of multi-peak loss indicates that the prepared material can adapt to more complex working environments and has stronger wave-absorbing performance. In addition, FeCoNi/SCA has a very low RL_min_ and a wide effective absorption bandwidth, which fully covers the X-band of electromagnetic waves, as well as most of the C-band and Ku-band.

**Fig. 3:**
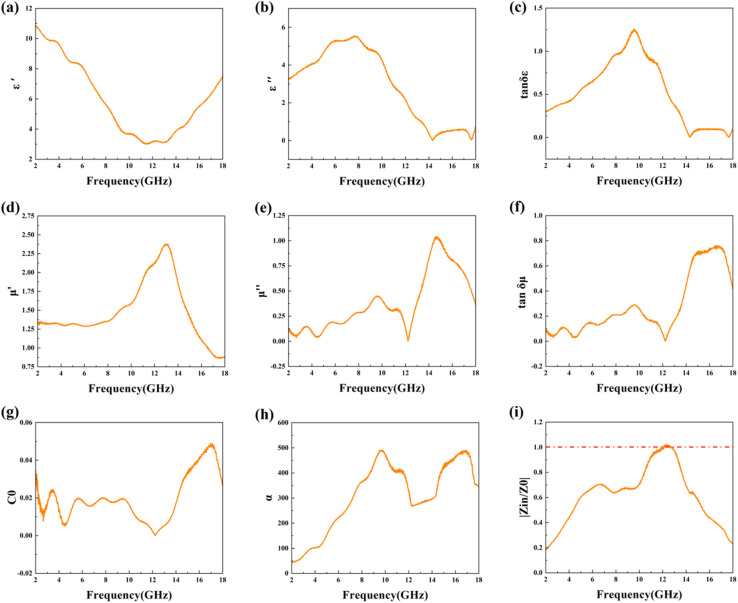
Real part (a) and imaginary part (b) of the complex permittivity and the real part (d) and imaginary part (e) of the complex permeability of FeCoNi/SCA at 2–18 GHz. The tan *δ*_*ε*_ (c) and tan *δ*_*µ*_ (f) values, (g) *C*_0_ values, (h) *α* values, and (i) |*Z*_in_/*Z*_0_| values of FeCoNi/SCA at 2–18 GHz.

To further analyze the wave-absorbing properties of the composite material, the electromagnetic parameters of FeCoNi/SCA were examined (Fig. 3).

Complex permittivity (*ε*_r_ = *ε*′ − j*ε*″) and complex permeability (*µ*_r_ = *µ*′ − j*µ*″) are core parameters for characterizing the microwave absorption performance of materials. The real parts (*ε*′, *µ*′) describe the ability of the material to store electric field energy and magnetic field energy under electromagnetic waves, respectively. The imaginary parts (*ε*″, *µ*″) correspond to the dissipation of electromagnetic energy. The electromagnetic properties of the material have a decisive effect on its wave-absorbing ability.^14^

Fig. 3(a and b) shows that the dielectric properties of the FeCoNi/SCA composite material are effectively regulated, which helps electromagnetic waves enter the material and be dissipated. The *ε*′ value generally decreases with increasing frequency but rises significantly in the high-frequency region (12–18 GHz). The *ε*″ value remains at a high level throughout the frequency band, showing that the material has strong dielectric loss capability, which supports its absorption performance. The dielectric loss behavior of the material mainly originates from the combined effect of interface polarization and dipole polarization. The heterogeneous interface between FeCoNi metal particles and starch-derived carbon (SCA) introduces a large number of defects and additional interfaces, which significantly increases interface polarization.

**Fig. 4:**
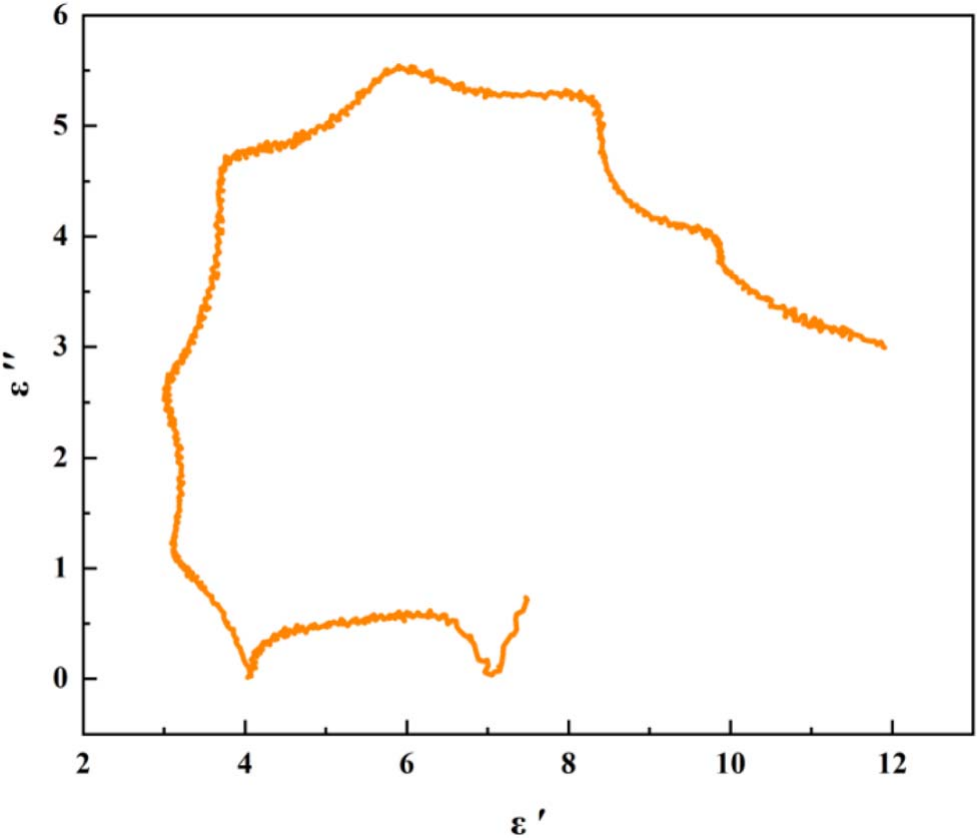
Cole–Cole curve of FeCoNi/SCA.

Furthermore, the uniform dispersion of *in situ* formed FeCoNi magnetic particles on the carbon matrix provides abundant polarization centers, enhancing dipole polarization and leading to more intense polarization relaxation. This relaxation behavior can be explained by the classical Debye theory (eqn (10)), and its structural origin can also be confirmed by Raman spectroscopy (Fig. 4).

Loss factors are another important indicator for analyzing electromagnetic parameters, including dielectric loss tangent and magnetic loss tangent. The calculation formulas are as follows:^15^4
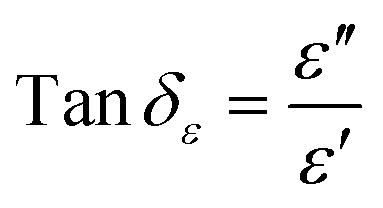
5
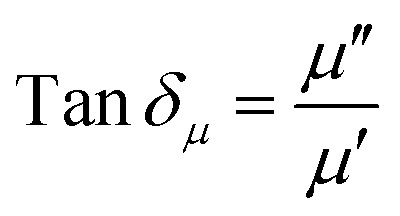


Fig. 3(c and f) shows the loss factor calculation results for FeCoNi/SCA. In the frequency range of 2–10 GHz, both tan *δ*_*ε*_ and tan *δ*_*µ*_ show an upward trend, and the former is significantly higher than the latter, which means that dielectric loss dominates in this frequency region. In frequency regions above 10 GHz, dielectric loss decreases while magnetic loss increases rapidly and becomes the main loss mechanism. Its loss mechanism transitions smoothly across the frequency domain, ensuring strong electromagnetic energy dissipation in the entire 2–18 GHz band.

From the curves, in most frequency regions, tan *δ*_*ε*_ and tan *δ*_*µ*_ values are of the same order of magnitude, and there is no extreme imbalance (*e.g.*, tan *δ*_*ε*_ >> tan *δ*_*µ*_). This indicates that dielectric loss and magnetic loss work together, helping FeCoNi/SCA achieve a wider wave-absorbing bandwidth. The synergy of dielectric and magnetic losses is crucial to obtaining high-performance wave-absorbing materials.

Dielectric polarization loss (including interfacial polarization and dipole polarization) and magnetic loss (including natural resonance, exchange resonance, and eddy current loss) are the two core loss systems for microwave absorption of the FeCoNi/SCA composite. To quantitatively distinguish their contributions to the total absorption loss, the loss tangent normalization method was adopted in this study, whose theoretical basis is derived from the basic definitions of electromagnetic parameters,^16–18^ and the formulas are as follows:6
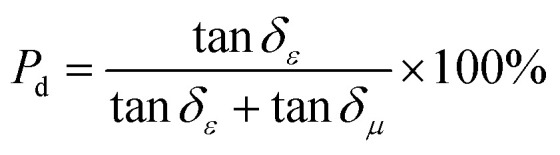
7
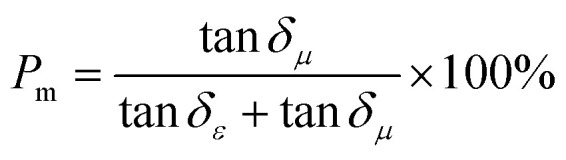
where *P*_d_ is the volume contribution ratio of dielectric polarization loss to the total loss, and *P*_m_ is the volume contribution ratio of magnetic loss to the total loss; tan *δ*_*ε*_ = *ε*″/*ε*′(*ε*′ is the real part of permittivity, *ε*″ is the imaginary part of permittivity), namely the dielectric loss tangent.

The calculated results based on the electromagnetic parameters of the optimal sample in this work at the best microwave absorption frequency band show that the microwave absorption loss of the FeCoNi/SCA composite is dominated by dielectric polarization loss (87.1%), supplemented by magnetic loss (12.9%).

Fig. 3(d and e) shows the complex permeability of FeCoNi/SCA. In general, magnetic loss mechanisms mainly include five types: hysteresis loss, domain wall resonance, natural resonance, exchange resonance, and eddy current loss. Hysteresis loss and domain wall loss occur in weak magnetic fields and low-frequency regions (MHz), so they can usually be excluded.

Therefore, only natural resonance, exchange resonance, and eddy current loss need to be considered. Eddy current loss originates from the energy dissipation caused by eddy currents in a changing magnetic field. It can be defined as:^19^

8 *C*_0_ = *µ*″(*µ*′)^−2^*f*^−1^ = 2π*µ*_0_*σd*^2^/3

where: *C*_0_ is a constant independent of *f*; *µ*_0_ is the vacuum permeability; and *σ* is the conductivity. If the magnetic loss is entirely caused by eddy current loss, the value of *C*_0_ remains constant regardless of frequency.

Fig. 3(g) shows the calculated *C*_0_ values of FeCoNi/SCA. It shows noticeable fluctuations in both the low-to-medium frequency range and in the high-frequency region (14–18 GHz). According to electromagnetic theory, magnetic loss in this range is dominated by exchange resonance, indicating that exchange resonance governs this frequency band.

This is consistent with the *µ*″ curve in Fig. 3(e), which shows stronger magnetic loss capability across the entire frequency band, related to the diverse magnetic loss mechanisms.

The ability of materials to dissipate electromagnetic energy can be quantitatively assessed by the attenuation coefficient *α*:^20^9
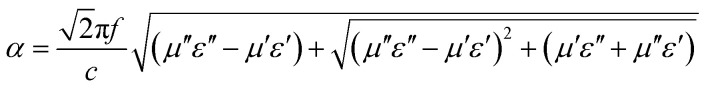


A higher *α* value represents better overall wave-absorbing ability. Fig. 3(h) shows the calculation results. Across the entire frequency band, *α* first increases rapidly, then decreases, and then rises again. Particularly in the range of 10–16 GHz, it rises sharply to a high level (peak value close to 500), indicating that electromagnetic waves can be efficiently converted into thermal energy and dissipated in this frequency band, with strong attenuation ability. This “high attenuation in the medium-to-high frequency band” feature aligns with the synergistic range of dielectric loss (peak near 10 GHz) and magnetic loss (enhanced after 12 GHz), ensuring strong absorption in this region and supporting the excellent wave-absorbing properties of FeCoNi/SCA.

Impedance matching ability is also an important factor influencing the wave-absorbing performance of materials. Fig. 3(i) shows the calculation results for impedance matching. When the impedance matching value (*Z*_in_/*Z*_0_) is equal to or close to 1, most incident electromagnetic waves can enter the interior of the absorbing material rather than being reflected at the interface between the absorber and free air. Therefore, for a wave-absorbing material to exhibit good performance, it must first meet the requirement of good impedance matching. It is evident that the value is very close to the ideal value of 1 in the frequency range of 10–14 GHz, indicating excellent impedance matching in this region, ensuring that electromagnetic waves can enter the interior of the material to the greatest extent.

Furthermore, the electromagnetic wave absorption performance is jointly determined by the impedance matching characteristic and the attenuation capability. The closer the impedance matching parameter |*Z*_in_/*Z*_0_| is to 1, the more favorable it is for electromagnetic waves to enter the interior of the material and reduce surface reflection.

On this basis, the attenuation constant *α* determines the loss capability of the material toward incident electromagnetic waves, and a larger *α* value generally indicates stronger dielectric loss, magnetic loss, and interfacial polarization relaxation effect.

When |*Z*_in_/*Z*_0_| is close to 1 and the attenuation constant *α* remains within a reasonable range, the material can not only ensure efficient incidence of electromagnetic waves but also achieve sufficient energy dissipation. The synergistic effect of the two factors finally realizes strong broadband absorption.

The dielectric loss behavior of materials can usually be interpreted using Debye theory:^21^10
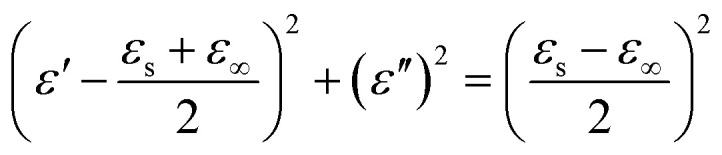
where *ε*_s_ and *ε*_∞_ are the static permittivity and high-frequency permittivity, respectively.

Dielectric loss can also be described using the Debye relaxation model (Cole–Cole) semicircle. With the real part of complex permittivity (*ε*′) on the horizontal axis and the imaginary part (*ε*″) on the vertical axis, the curve shape reflects the polarization relaxation behavior and conductivity characteristics of the material.

The semicircle that appears in the plot is called the Cole–Cole semicircle. Each Cole–Cole semicircle indicates that a Debye relaxation process occurs in the material.^22^

Fig. 4 shows the Cole–Cole relationship curve of FeCoNi/SCA. It can be seen that the curve presents several relatively complete and smooth Cole–Cole semicircles, indicating that multiple Debye relaxation processes occur. The relatively large number of complete Cole–Cole semicircles reflects that polarization relaxation behavior takes place repeatedly and in a more complete manner. Such relaxation typically originates from orientational polarization of dipoles inside the material or interface polarization at heterogeneous interfaces.

The presence of a complete semicircle suggests that the sample has a high density of defects and abundant heterogeneous interfaces (such as at the carbon layer-metal particle interface or the magnetic-carbon composite interface). These regions can trap charges to form dipoles, which undergo relaxation under an external electric field, resulting in significant polarization loss.

### 3.2 Raman spectroscopy analysis

The structural information of carbon was further analyzed using Raman spectroscopy, which is often used to examine carbon atom states and the graphitization degree of carbon materials. In Raman spectroscopy, the D-band and G-band are characteristic spectral features of carbon materials and are widely used to investigate carbon-based structures. The D-band is usually around 1350 cm^−1^, associated with defects in carbon (such as missing atoms or distorted atomic positions). The G-band is typically around 1590 cm^−1^, caused by sp^2^-hybridized carbon atoms and representing the planar vibration of the graphite lattice.

Researchers often characterize the defect level (or graphitization degree) of carbon by calculating the intensity ratio of the D-band to the G-band (*I*_D_/*I*_G_).^23^ In general, a higher *I*_D_/*I*_G_ ratio indicates lower graphitization, more defects, and a higher degree of disorder in the material.

**Fig. 5:**
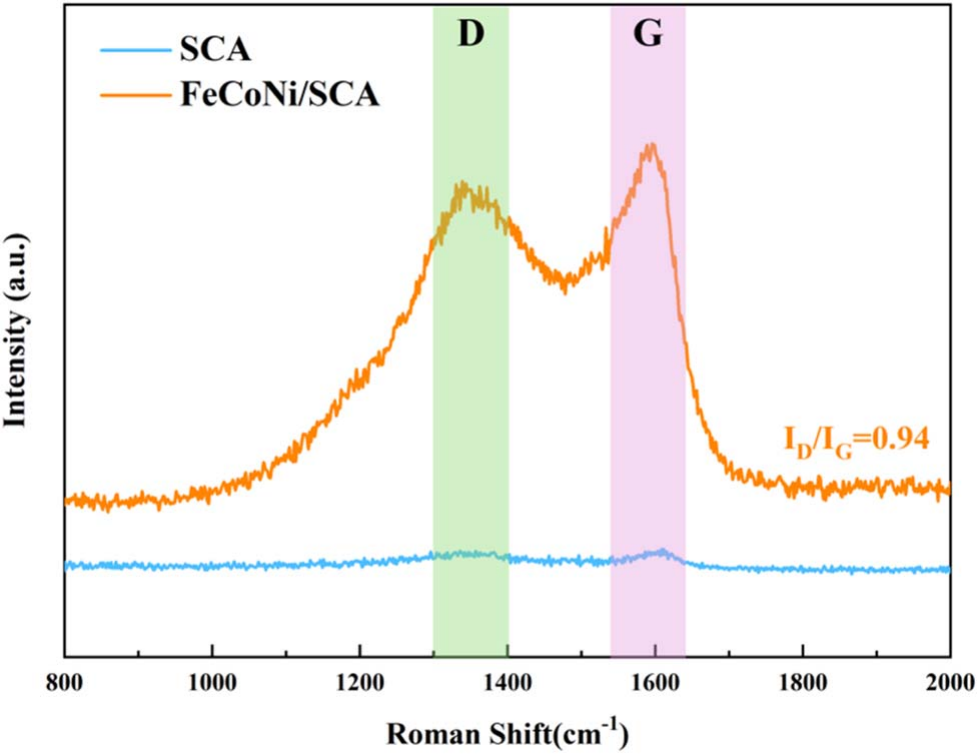
Raman analysis of FeCoNi/SCA.

Fig. 5 shows that the SCA sample without FeCoNi doping has no obvious D-band or G-band and appears almost as a straight line. However, the FeCoNi/SCA sample after doping has two clear peaks at 1350.0 cm^−1^ and 1590.0 cm^−1^, representing the typical Raman signatures of disordered carbon materials, namely the “D peak” and “G peak.” This demonstrates that the calcination process successfully converted cassava starch into a carbon matrix.

The appearance of the D-band may be linked to the reduction of magnetic particles (such as Fe^3+^, Co^2+^, Ni^2+^) during carbonization and the gas produced during this process. These factors can introduce defects, and the nitrogen-doped carbon layer can also increase the defect level, which plays an important role in regulating dielectric properties. In an electromagnetic field, abundant defects can act as polarization centers and enhance the wave-absorbing properties of the material. The *I*_D_/*I*_G_ value is 0.94, close to 1, indicating a large number of structural defects or disordered regions (such as interfaces between FeCoNi particles and the carbon matrix, or edge carbon of porous structures). These defects provide numerous polarization centers for interface and dipole polarization, which is one of the key contributors to the strong dielectric loss of the material.

The *I*_D_/*I*_G_ value of the FeCoNi/SCA sample is 0.94, indicating the presence of moderate structural defects in the material. It should be noted that the *I*_D_/*I*_G_ ratio alone cannot be directly regarded as excessively high defect density, and its effect on electromagnetic properties should be comprehensively evaluated by combining the integrity of the conductive network and the polarization behavior. On the one hand, a proper amount of defects can act as favorable polarization centers, providing more sites for interfacial polarization and dipole polarization, thus enhancing dielectric loss. On the other hand, overly dense defects will destroy the continuous conductive network of the carbon matrix, leading to decreased conductivity, which is unfavorable for the effective attenuation of electromagnetic waves.

Combined with the electromagnetic performance data in this work, the *I*_D_/*I*_G_ ratio of 0.94 just strikes a balance between the two aspects: it introduces sufficient polarization centers through moderate defects to improve dielectric loss, while retaining the continuous conductive network of the carbon matrix to ensure good conductivity. This provides structural and performance guarantees for the excellent microwave absorption properties.

### 3.3 Hysteresis loop analysis

The magnetic properties of samples SCA and FeCoNi/SCA were characterized using VSM.

**Fig. 6:**
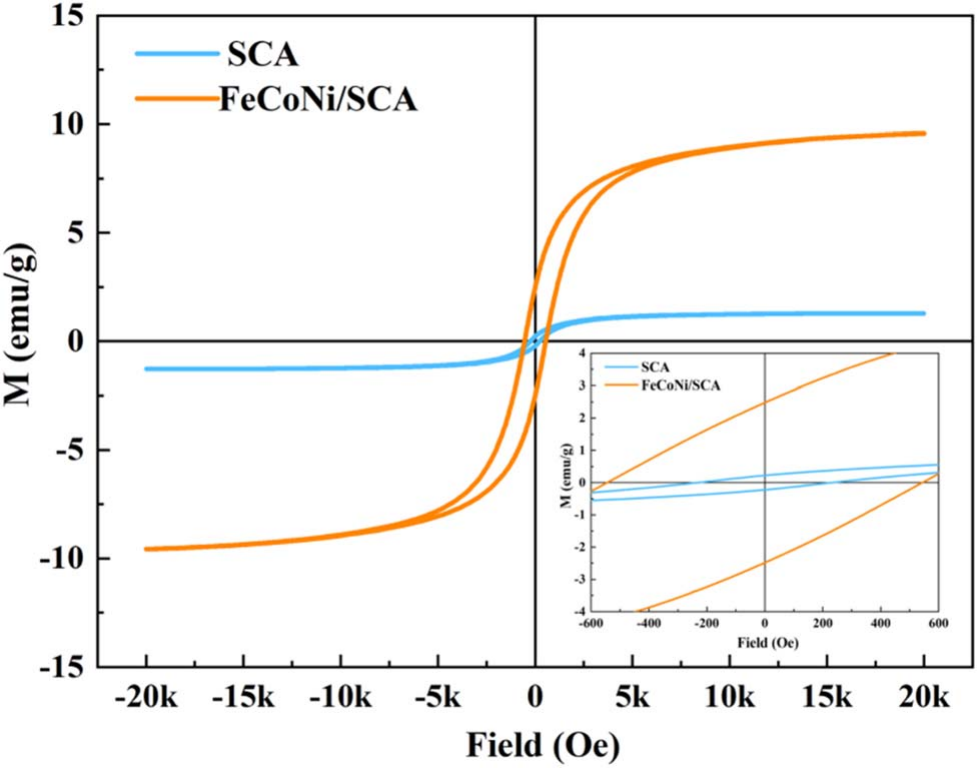
Magnetic hysteresis loops of SCA and FeCoNi/SCA.

From the room-temperature hysteresis loops shown in Fig. 6, it can be seen that the FeCoNi/SCA sample exhibits ferromagnetic behavior with clear hysteresis.^24^ Compared with SCA, the FeCoNi/SCA sample shows more pronounced hysteresis behavior, indicating that magnetic particles were successfully loaded on the SCA surface.

Under a test magnetic field of 20 k Oe, the saturation magnetization (*M*_S_ = 9.56 emu g^−1^) of FeCoNi/SCA is much higher than that of SCA (*M*_S_ = = 1.28 emu g^−1^), mainly due to the high saturation magnetization of FeCoNi particles and their uniform and dense distribution on the SCA surface. Meanwhile, coercivity (*H*_c_) is another key parameter identified from the hysteresis loop. The coercivity of FeCoNi/SCA (*H*_c_ = 534 Oe) is significantly higher than that of SCA (*H*_c_ = 231 Oe).

The increase in *H*_c_ mainly originates from the magnetocrystalline anisotropy provided by FeCoNi particles, which can enhance hysteresis loss and contribute to magnetic loss support for the material's electromagnetic wave absorption behavior.

### 3.4 Phase composition analysis

**Fig. 7:**
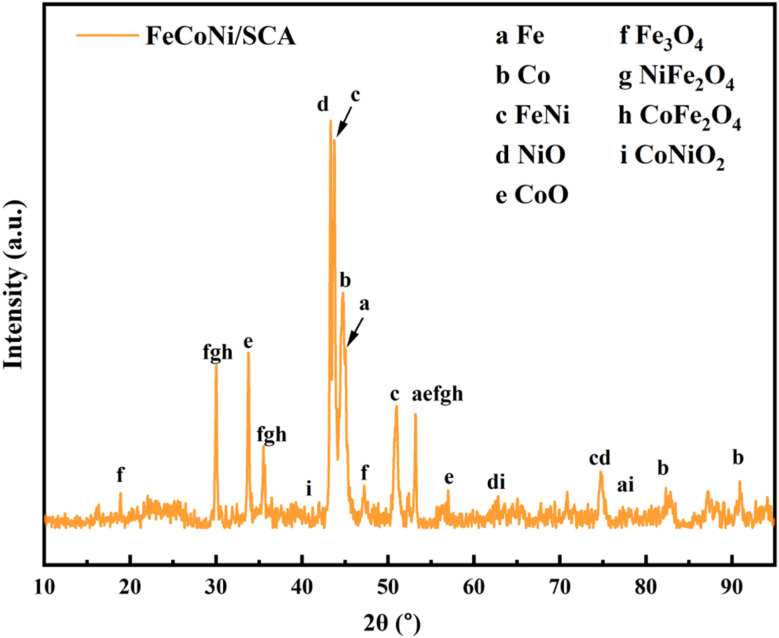
XRD pattern of FeCoNi/SCA.

The phase composition of FeCoNi/SCA can be examined using XRD diffraction analysis (Fig. 7).

XRD results show that FeCoNi/SCA displays multiple sharp diffraction peaks in the 2*θ* range of 5–95°, indicating that the material has a well-crystallized multi-phase composite structure. By phase identification and comparison with JCPDS standard cards, the characteristic diffraction peaks can be assigned as follows:

Metallic phase: the diffraction peaks at 2*θ* = 44.7°, 51.8°, and 79.2° correspond to the (110), (200), and (211) crystal planes of metallic Fe (PDF#88-2324), respectively. The diffraction signals at 2*θ* = 41.7°, 44.8°, and 47.5° match well with the (100), (002), and (101) crystal planes of metallic Co (PDF#05-2727), indicating that part of the metal remains in elemental form.

Alloy phase: the broadened peak near 2*θ* = 43.5° can be assigned to the FeNi alloy phase (PDF#47-1405). Its peak position shift relative to the pure metal phase indicates the formation of an Fe–Ni solid solution, accompanied by atomic interactions and lattice distortion.

Oxide phase: several oxide phases are evident in the diffraction pattern. The peaks at 2*θ* = 37.2° and 62.8° correspond to the (111) and (220) planes of NiO (PDF#44-1159). The characteristic peak at 2*θ* = 36.5° matches the (111) plane of CoO (PDF#75-0419), indicating that part of the metal was oxidized during synthesis. In addition, characteristic peaks of NiFe_2_O_4_ (PDF#86-2267) and CoFe_2_O_4_ (PDF#79-1744) appear near 2*θ* = 35.3° and 35.5°, respectively. Combined with the magnetite Fe_3_O_4_ signal (PDF#99-0073) at 2*θ* = 30.1° (220), these results confirm the presence of various spinel-type ferrites. These spinel ferrites are efficient magnetic loss media, and their coexistence can enhance the overall magnetic loss capacity of the material through synergistic mechanisms.

There may also be a rock-salt type CoNiO_2_ phase, with typical peaks around ∼36.5° and ∼42.5°, although these may overlap with ferrite and alloy phase peaks.

Amorphous carbon matrix: the slight bulge of the XRD baseline in the range of 20–30° indicates the presence of an amorphous carbon phase formed by starch carbonization, and its composite structure with metal/oxide nanocrystals can be inferred from the broadening of certain peaks.

In summary, XRD analysis demonstrates that a magnetic multiphase system consisting of metallic elements, alloys and various spinel ferrites has been successfully constructed in the composite after high-temperature calcination. While such a multiphase structure enhances magnetic loss, it also gives rise to increased electrical conductivity: an excessively high content of metallic and alloy phases will lead to a significant increase in permittivity, a reduction in impedance matching, and intensified reflection of electromagnetic waves on the material surface, which is detrimental to the improvement of microwave absorption performance. The core design strategy of this work is to balance and regulate this contradiction. On the one hand, the coexistence of metallic, alloy and oxide phases is fully utilized to remarkably strengthen the magnetic loss of the material, providing core support for high-efficiency microwave absorption. On the other hand, by strictly controlling the total dosage of magnetic phases and employing the three-dimensional porous structure of starch-based carbon aerogel to realize uniform dispersion of each phase, the excessive agglomeration of conductive phases into a continuous highly conductive network is avoided.

By tuning the content and dispersion state of the multiphases, the effective enhancement of magnetic loss is guaranteed and favorable impedance matching is achieved at the same time. This allows electromagnetic waves to smoothly enter the material interior and be fully dissipated, thus finally delivering excellent microwave absorption performance.

### 3.5 Surface morphology analysis

Starch is a polysaccharide polymerized from glucose molecules, mainly composed of amylose and amylopectin.^25^ When starch granules are heated in water at a certain temperature, they swell, accompanied by the exudation of amylose, destruction of the ordered crystalline structure, and rupture and collapse of starch granules. This process is called starch gelatinization.^26^ During gelatinization, starch granules absorb water and swell, and their molecular chains stretch and entangle in a double helix to form a gel network. During the subsequent low-temperature aging stage, adjacent molecules recombine through hydrogen bonding, gradually forming dense microbundles and ultimately a stable hydrogel. After freeze-drying, an aerogel with a three-dimensional porous network structure is obtained. The microstructure of FeCoNi/SCA was characterized using scanning electron microscopy (SEM), and the results are presented as follows.

**Fig. 8:**
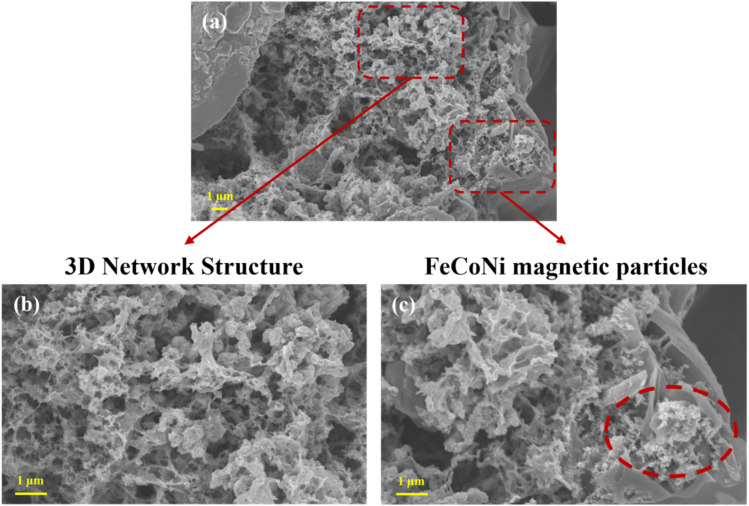
Surface morphology of FeCoNi/SCA at 5000× magnification and microstructure at 10 000× magnification, (a) FeCoNi magnetic particles, (b) 3D network structure, (c) FeCoNi magnetic particles.

As shown in Fig. 8(b), after carbonization, the aerogels retain the porous features of their xerogel precursors. The aerogel pores facilitate the entry of electromagnetic waves into the material, balancing impedance, and also create pathways for multiple reflections and scattering, improving electromagnetic wave attenuation. On one hand, the pore size distribution is uniform and orderly, and the increased pore density forms a clearer multi-level pore system, greatly increasing the specific surface area. On the other hand, the pores exhibit irregular shapes, retaining some large open-cell structures while generating numerous fine closed-cell pores, forming a multi-scale pore system with both open and closed cells.

By *in situ* introducing FeCoNi magnetic particles into the three-dimensional porous network of starch aerogels, structural and functional synergistic regulation is achieved. Starch is carbonized, transforming from a polysaccharide to conductive a carbon aerogel skeleton (SCA). FeCoNi precipitates are reduced to form FeCoNi magnetic nanoparticles. As shown in Fig. 8(c), FeCoNi nanoparticles are uniformly loaded on the fiber surface, consistent with the XRD results. Larger magnetic nanoparticles form at this stage, enhancing the Fe_3_O_4_ diffraction peak in XRD and creating a rich three-dimensional network together with the aerogel matrix. This structure promotes multiple reflection and scattering losses of electromagnetic waves inside the material, enhancing the overall microwave absorption performance.

**Fig. 9:**
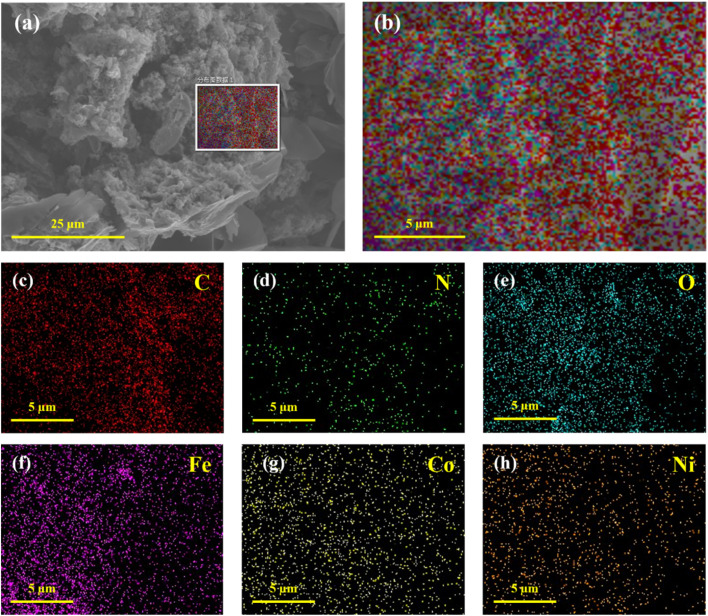
(a) FeCoNi/SCA at 3000× magnification, (b) elemental mapping, (c) C element, (d) N element, (e) O element, (f) Fe element, (g) Co element, (h) Ni element.

To further verify the surface elemental composition of the FeCoNi/SCA composite material, high-resolution elemental mapping was carried out *via* transmission electron microscopy (Fig. 9).

As shown in Fig. 9, the material contains six elements: C, N, O, Fe, Co, and Ni. Each element is uniformly distributed, indicating that magnetic particles are evenly dispersed on the carbon skeleton without obvious agglomeration.

Carbon content is the highest, with a continuous and uniform distribution, confirming that the material is mainly composed of carbon as the structural framework of the aerogel. Oxygen content is also high, mainly originating from the following sources: hydroxyl groups in the active FeCoNi component, residual hydroxyl groups, ether bonds, and other oxygen-containing functional groups in the starch-based biomass-derived carbon skeleton, and sulfate ions (SO_4_^2−^) introduced from precursor salts.

The distributions of Fe, Co, and Ni are relatively uniform, further confirming the successful synthesis of FeCoNi alloy and showing good compositional uniformity.

**Fig. 10:**
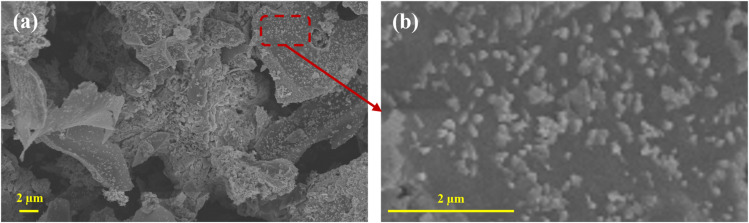
Micromorphologies of FeCoNi/SCA at magnifications of (a) 3000× and (b) 198 00×.

To further reveal the dispersion state and particle size distribution of FeCoNi nanoparticles in the carbon aerogel matrix, FeCoNi/SCA was characterized by scanning electron microscopy (SEM) at different magnifications (Fig. 10).

As can be seen from Fig. 10(a), FeCoNi/SCA exhibits a three-dimensional porous skeleton structure composed of carbon aerogel, and FeCoNi particles are uniformly distributed on the surface without obvious agglomeration.

Fig. 10(b) further clearly shows that FeCoNi particles are homogeneously dispersed on the carbon aerogel surface with no large-scale aggregation between particles, which provides a favorable structural basis for the subsequent optimization of electromagnetic properties.

Particle size analysis of the particles on the surface of FeCoNi/SCA was carried out using ImageJ software.

**Fig. 11:**
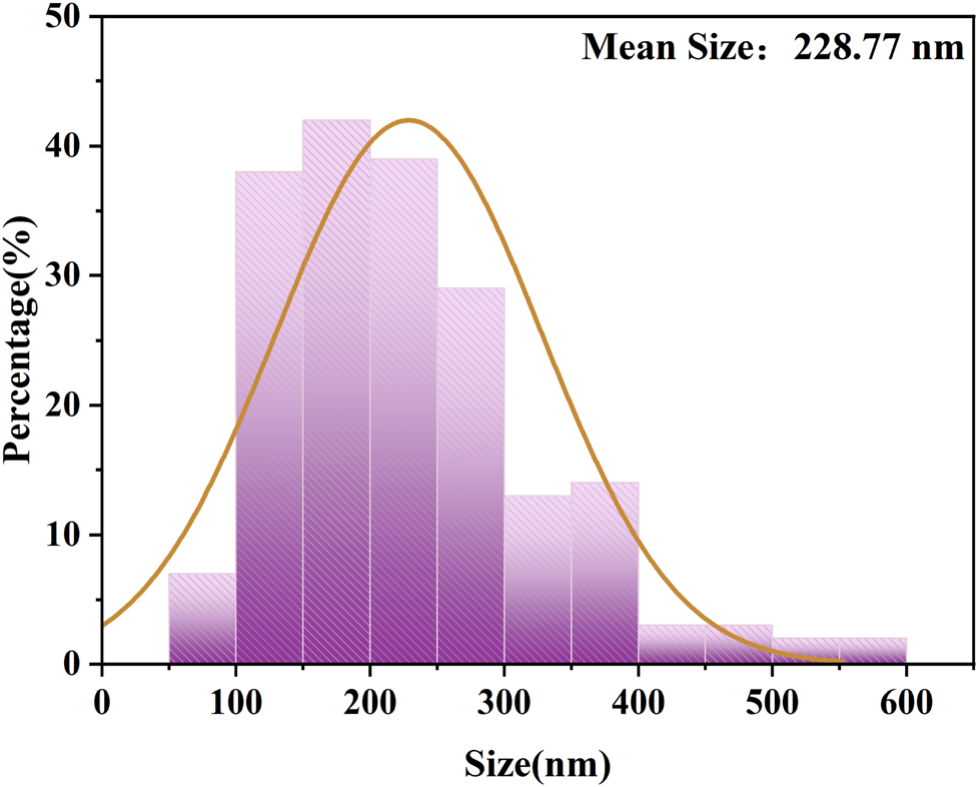
Particle size analysis of the surface of FeCoNi/SCA.

The results indicate that the average particle size on the surface is 228.77 nm (Fig. 11).

### 3.6 Surface chemical composition analysis

**Fig. 12:**
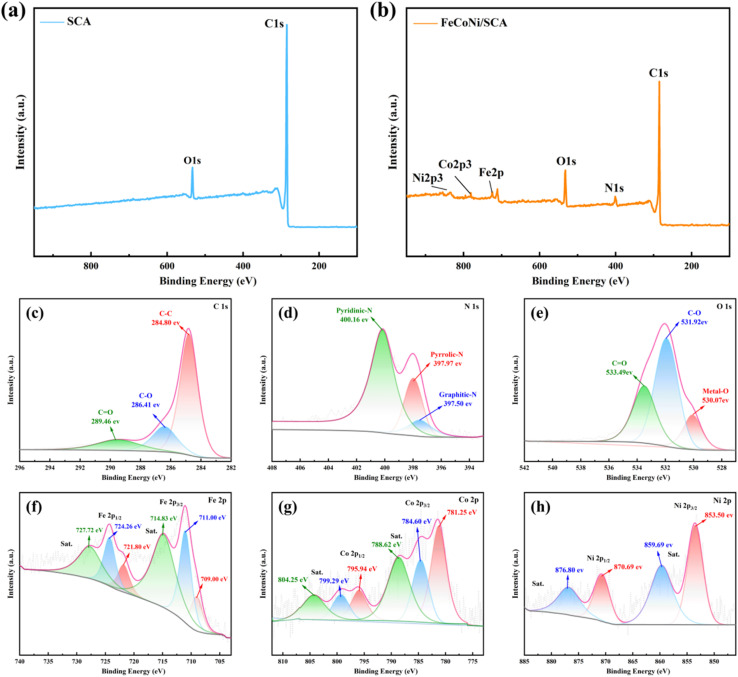
(a) XPS full spectrum of SCA, (b) XPS full spectrum of FeCoNi/SCA, high-resolution XPS spectra of FeCoNi/SCA: (c) C1s, (d) N1s, (e) O1s, (f) Fe2p, (g) Co2p, (h) Ni2p.

Fig. 12(a and b) show the full spectra of SCA and FeCoNi/SCA, and the high-resolution spectra of C 1s, N 1s, O 1s, Fe 2p, Co 2p and Ni 2p were peak-fitted. The detailed fitting parameters (binding energy, functional group, percentage of peak area, full width at half maximum and binding energy error) are summarized in Table 1.

**Table 1:** Fitting parameters of high-resolution XPS spectra

Spectrum	Binding Energy (eV)	Chemical species	Peak area ratio (%)	FWHM (eV)	Binding energy error (eV)
C 1s	284.80	C–C	66.45	1.40	±0.03
C 1s	286.41	C–O	19.24	1.92	±0.04
C 1s	289.46	C=O	14.31	3.35	±0.05
N 1s	397.50	Graphitic-*N*	8.49	1.94	±0.04
N 1s	397.97	Pyrrolic-*N*	20.64	1.50	±0.03
N 1s	400.16	Pyridinic-*N*	70.87	2.02	±0.04
O 1s	530.07	Metal-O	14.85	1.53	±0.05
O 1 s	531.92	C–O	54.54	1.92	±0.04
O 1s	533.49	C=O	30.61	1.92	±0.05
Fe 2p	709.00	Fe 2p_3/2_(Fe^2+^)	8.02	2.40	±0.05
Fe 2p	711.00	Fe 2p_3/2_(Fe^3+^)	21.50	2.40	±0.05
Fe 2p	714.83	Fe 2p_3/2_ Sat.	36.63	4.86	±0.05
Fe 2p	721.80	Fe 2p_1/2_(Fe^2+^)	6.42	2.40	±0.05
Fe 2p	724.26	Fe 2p_1/2_(Fe^3+^)	11.37	2.40	±0.05
Fe 2p	727.72	Fe 2p_1/2_ Sat.	16.06	4.86	±0.05
Co 2p	781.25	Co 2p_3/2_(Co^3+^)	28.70	3.35	±0.05
Co 2p	784.60	Co 2p_3/2_(Co^2+^)	18.02	3.50	±0.05
Co 2p	788.62	Co 2p_3/2_ Sat.	26.83	4.79	±0.05
Co 2p	795.94	Co 2p_1/2_(Co^3+^)	8.87	2.50	±0.05
Co 2p	799.29	Co 2p_1/2_(Co^2+^)	7.29	3.50	±0.05
Co 2p	804.25	Co 2p_1/2_ Sat.	10.29	4.79	±0.05
Ni 2p	853.50	Ni 2p_3/2_(Ni^2+^)	36.21	3.37	±0.05
Ni 2p	859.69	Ni 2p_3/2_ Sat.	32.06	4.79	±0.05
Ni 2p	870.69	Ni 2p_1/2_(Ni^2+^)	16.83	3.37	±0.05
Ni 2p	876.80	Ni 2p_1/2_ Sat.	14.90	4.79	±0.05

XPS full-spectrum analysis shows that SCA presents two characteristic peaks at 285.18 eV and 533.21 eV, corresponding to C1s and O1s, respectively. The FeCoNi/SCA composite material contains C, N, O, Fe, Co, and Ni elements simultaneously. Fig. 12(c) shows the high-resolution C1s energy spectrum of the composite material, which displays three characteristic peaks at binding energies of 284.80, 286.41, and 289.46 eV, corresponding to C–C, C–O, and C=O bonds, respectively.

In Fig. 12(d), the peaks with binding energies of 397.50, 397.97, and 400.16 eV correspond to graphitic nitrogen, pyrrolic nitrogen, and pyridinic nitrogen (graphitic-*N*, pyrrolic-*N*, and pyridinic-*N*), respectively. The introduction of these nitrogen species is beneficial for enhancing the electromagnetic wave absorption of the composite material. Specifically, the N-doped carbon coating can affect the response of the composite material to EMW in two ways: graphitic-*N* introduces a large number of p-electrons into the delocalized π-bond system of graphitic carbon, improving electrical conductivity and enhancing conduction loss; meanwhile, pyridinic-*N* and pyrrolic-*N* introduce lattice defects that hinder carrier migration, thereby increasing dipole polarization loss.

The O1s spectrum in Fig. 12(e) can be fitted into three peaks at 530.07 eV, 531.92 eV, and 533.49 eV, corresponding to metal–O bonds, C–O bonds, and C=O bonds, respectively. The metal–O bonds originate from the inherent structure of FeCoNi in the precursor and its coordination with oxygen-containing functional groups on the carbon matrix. After high-temperature pyrolysis, metal ions are reduced to metal alloys (such as FeNi), which grow on the coating surface. At the same time, oxygen-containing functional groups can serve as polarization centers, improving dipole polarization and relaxation, and thus weakening the incident EMW.

As shown in Fig. 12(f), in the Fe 2p spectrum of FeCoNi/SCA, the characteristic peaks at 711.00 eV and 724.26 eV correspond to Fe 2p_3/2_ and Fe 2p_1/2_, respectively. Their binding energy position indicates that Fe mainly exists in the +3 valence state (Fe^3+^). Satellite peaks at 714.83 and 727.72 eV are also observed, consistent with reported features of trivalent iron oxides/hydroxides.

In the Co 2p spectrum in Fig. 12(g), the main peaks of Co 2p_3/2_ and Co 2p_1/2_ are located at 781.25 eV and 795.94 eV, accompanied by satellite peaks at 784.60 and 799.29 eV. The binding energy position and satellite peak structure are typical features of Co^2+^ species, indicating that cobalt mainly exists in the +2 valence state, which may originate from phases such as CoO or cobalt iron oxides.

In the Ni 2p spectrum in Fig. 12(h), the main peaks of Ni 2p_3/2_ and Ni 2p_1/2_ appear at 853.50 eV and 870.69 eV, and satellite peaks are located at 859.69 eV and 876.80 eV, respectively, indicating that both Ni^2+^ and Ni^3+^ exist in the sample, forming compounds such as NiO and FeNi. In addition, the Fe, Co, and Ni 2p spectra all present asymmetric peak shapes, indicating that these elements exist in multiple chemical states in the material.

It is worth noting that the ferrite phase in the material has a cubic spinel structure. Typically, Co and Ni ions occupy about 50% of the B-site in their ion distribution as divalent metal cations. The fitting results suggest that nickel ferrite and cobalt ferrite are generated in the sample, both presenting an inverse spinel structure. This observation is consistent with the results of the fitting process above. The coexistence of multiple magnetic components significantly enhances the magnetic loss capacity of the material, thereby improving its overall wave-absorbing performance.

### 3.7 Wave-absorbing mechanism of FeCoNi/starch-based carbon composite aerogels

The excellent electromagnetic wave absorption performance of the FeCoNi/SCA composite originates from the synergistic effect of multiple loss mechanisms induced by its biomass-derived carbon–magnetic composite structure. Combined with the experimental characterization results and theoretical analyses reported in ref. 27–29, the loss mechanism can be interpreted as follows:

(1) The carbon aerogel formed from cassava starch after high-temperature carbonization possesses a continuous three-dimensional porous carbon skeleton, which facilitates electron migration inside the material. Under an alternating electromagnetic field, free electrons undergo directional migration and multiple scattering in the carbon skeleton, converting electromagnetic energy into thermal energy and thus generating conductive loss. Meanwhile, the ultrahigh porosity of the carbon aerogel causes multiple reflections and refractions of electromagnetic waves inside the material, prolonging the propagation path and enhancing the interaction between electromagnetic waves and the conductive skeleton, further improving the energy dissipation efficiency.

(2) FeCoNi magnetic particles are uniformly dispersed and loaded on the surface of the carbon aerogel skeleton, forming numerous FeCoNi/carbon heterointerfaces between the two components. Due to the electron transfer tendency between FeCoNi and carbon materials, uneven charge distribution occurs at the interfaces, forming local built-in electric fields. Under the alternating electromagnetic field, these bound charges cannot respond to the external field in time, thus triggering interfacial polarization relaxation and significantly enhancing dielectric loss.

(3) Carbon defects (such as edge defects and vacancies) are inevitably formed during the carbonization of starch, and the introduction of FeCoNi particles induces local lattice distortion. These defects and distorted regions can act as polarization centers to trap charges and form dipoles. Under the alternating electric field, these dipoles undergo repeated orientation and relaxation, converting electromagnetic energy into thermal energy and thus enhancing dielectric loss. In addition, residual oxygen-containing functional groups (such as C–OH and C=O) on the carbon aerogel surface also introduce additional dipole polarization centers, which act synergistically with defect-induced dipole polarization and broaden the frequency response range of dielectric loss, providing important support for broadband absorption of the material.

(4) FeCoNi magnetic particles can produce various magnetic loss mechanisms under alternating magnetic fields, including natural resonance, exchange resonance, and hysteresis loss, which form a synergistic effect with the dielectric loss of the carbon aerogel. On the one hand, the introduction of magnetic particles optimizes the complex permeability of the material, making the input impedance of the material better match the impedance of free space, reducing the reflection of electromagnetic waves on the material surface and allowing more electromagnetic waves to enter the material for dissipation. On the other hand, magnetic loss and dielectric loss complement each other in the frequency domain: magnetic loss contributes significantly at low and medium frequencies, while dielectric loss is prominent at medium and high frequencies, jointly constructing a broadband and strong absorption loss system.

**Fig. 13:**
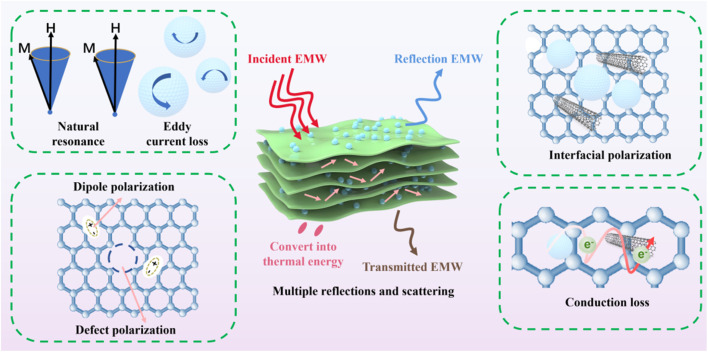
Schematic illustration of the microwave absorption mechanism of FeCoNi/SCA, involving conductive loss, interfacial polarization, dipole polarization, magnetic loss, and multiple reflections/scattering.

In summary, the microwave absorption mechanism of the FeCoNi/SCA composite takes the three-dimensional carbon aerogel conductive network as the matrix, constructs multiple heterointerfaces and magnetic loss centers by introducing FeCoNi particles, and utilizes defect-induced dipole polarization, forming a synergistic loss mechanism of conductive loss, interfacial polarization, dipole polarization, and magnetic loss. Such a multi-mechanism synergistic design strategy provides a theoretical basis for the development of high-performance biomass-derived microwave absorption materials (Fig. 13).

### 3.8 Comparison of microwave absorption performance

**Fig. 14:**
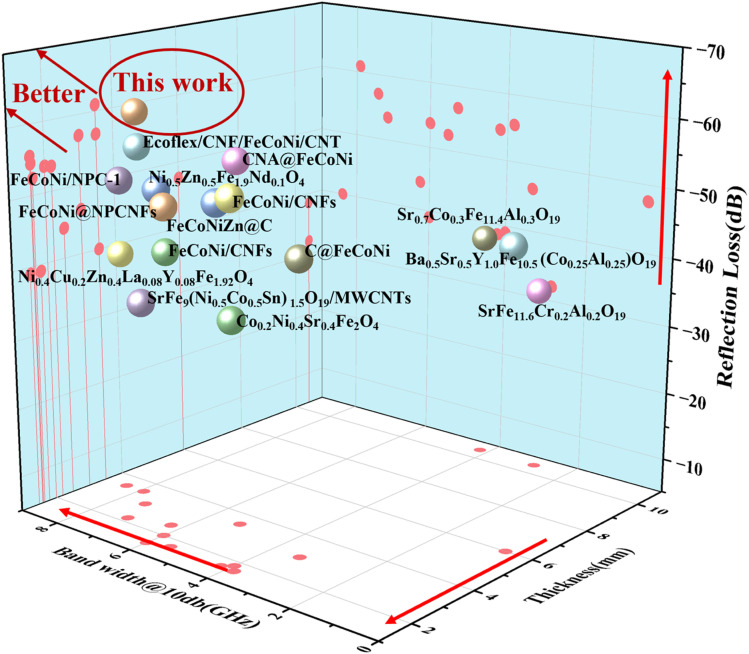
3D comparison of the minimum reflection loss, absorption bandwidth, and thickness for this work and recent literature (the closer to the upper left corner of the cube, the better the material absorbs waves).

In recent years, numerous researchers have carried out studies on microwave absorption materials. We have compared the present work with some representative ones, as shown in Fig. 14:

It is evident that the FeCoNi/SCA prepared in this study has the advantages of high reflection loss, low thickness, and high bandwidth.

#### 3.8.1 Comparison with representative microwave absorbing materials

In recent years, a variety of ferrite-based microwave absorption materials have been investigated.^30–36^ Pure ferrite-based absorbers (Co_0.2_Ni_0.4_Sr_0.4_Fe_2_O_4_, SrFe_11.6_Cr_0.2_Al_0.2_O_19_, Sr_0.7_Co_0.3_Fe_11.4_Al_0.3_O_19_) have achieved remarkable breakthroughs in regulating magnetocrystalline anisotropy and realizing high reflection loss through precise ion substitution strategies, providing important theoretical and experimental support for the component design of microwave absorption materials (Table 2).

**Table 2:** Comparison with representative microwave absorbing materials

Materials	RL_min_ (dB)	EAB (GHz)	Thickness (mm)	Ref.
Co_0.2_Ni_0.4_Sr_0.4_Fe_2_O_4_	−39	4.0	1.8	30
SrFe_9_(Ni_0.5_Co_0.5_Sn)_1.5_O_19_/MWCNTs	−39	6.0	1.5	31
Ni_0.4_Cu_0.2_Zn_0.4_La_0.08_Y_0.08_Fe_1.92_O_4_	−40	8.4	Not disclosed	32
Ni_0.5_Zn_0.5_Fe_1.9_Nd_0.1_O_4_	−53.72	6	2	33
SrFe_11.6_Cr_0.2_Al_0.2_O_19_	−32.2	2.81	10.5	34
Sr_0.7_Co_0.3_Fe_11.4_Al_0.3_O_19_	−38.5	4.2	10.5	35
Ba_0.5_Sr_0.5_Y_1.0_Fe_10.5_(Co_0.25_Al_0.25_)O_19_	−47.58	0.609	6	36
**FeCoNi/SCA**	**−60.89**	**7.91**	**3.5**	**This work**

Rare-earth-doped ferrite absorbers (Ni_0.4_Cu_0.2_Zn_0.4_La_0.08_Y_0.08_Fe_1.92_O_4_, Ni_0.5_Zn_0.5_Fe_1.9_Nd_0.1_O_4_) have realized strong reflection loss in specific frequency bands, confirming the unique advantages of rare-earth doping in enhancing magnetic anisotropy and optimizing impedance matching, and offering key guidance for the component design of high-loss microwave absorption materials.

Carbon-based composite and multication-doped ferrite absorbers (SrFe_9_(Ni_0.5_Co_0.5_Sn)_1.5_O_19_/MWCNTs, Ba_0.5_Sr_0.5_Y_1.0_Fe_10.5_(Co_0.25_Al_0.25_)O_19_) present innovative structural design concepts. The former broadens the effective absorption bandwidth efficiently by combining ferrite with carbon nanotubes, while the latter achieves high reflection loss through multi-component doping. These excellent studies have provided important references for the present work (Table 3).

**Table 3:** Comparison with FeCoNi-based biomass-derived carbon absorbers

Materials	RL_min_ (dB)	EAB (GHz)	Thickness (mm)	Ref.
FeCoNi@NPCNFs	−52.36	5.52	1.66	37
FeCoNi/CNFs	−44.3	6.25	2.5	38
FeCoNi/NPC-1	−53.50	7.15	2.19	39
FeNiCo/CNFs	−55.5	3.84	1.6	40
FeCoNiZn@C	−54.46	4.19	1.62	41
CNA@FeCoNi	−56.7	5.28	Not disclosed	42
C@FeCoNi	−46.4	3.35	3	43
Ecoflex/CNF/FeCoNi/CNT	−57	7.41	3	44
**FeCoNi/SCA**	**−60.89**	**7.91**	**3.5**	**This work**

#### 3.8.2 Comparison with FeCoNi-based biomass-derived carbon absorbers

In recent years, FeCoNi-based biomass carbon microwave absorption materials have attracted widespread attention, fully confirming the application potential of FeCoNi/biomass-derived carbon composite materials in the field of microwave absorption.

Although various related studies on FeCoNi-based biomass carbon microwave absorption materials have been reported,^37–44^ the FeCoNi/SCA prepared in this work still exhibits significant advantages in terms of comprehensive microwave absorption performance.

In this study, FeCoNi alloy/oxide phases were introduced into a starch-based biomass-derived carbon skeleton to construct a composite absorber with both magnetic–dielectric synergistic loss and a lightweight porous structure. While maintaining a high reflection loss (RL_min_ = −60.89 dB), the effective absorption bandwidth is broadened to 7.91 GHz, showing overall characteristics of high loss and broad bandwidth. Using widely available, low-cost, and biodegradable cassava starch as the carbon source significantly reduces the reliance on expensive carbon materials such as graphene and carbon nanotubes, and overcomes the high-cost drawback of rare-earth-doped systems. Compared with carbon nanotube composite systems, the three-dimensional porous structure of starch-based carbon aerogel can effectively suppress the agglomeration of conductive phases, promote more sufficient interfacial polarization, and achieve higher magnetic–dielectric synergistic loss efficiency, further optimizing the microwave absorption performance.

## 4 Conclusions

In this study, cassava starch acted as the carbon precursor. Starch-based aerogel/FeCoNi composite absorbing materials (FeCoNi/SA) were produced by a sol–gel process followed by freeze-drying, and starch-based carbon aerogel/FeCoNi composites (FeCoNi/SCA) were obtained through high-temperature carbonization. After carbonization, a three-dimensional porous carbon aerogel network was formed, with FeCoNi nanoparticles evenly distributed in the carbon matrix. Heterogeneous interface polarization and metal ion dipole polarization produced relaxation loss under an electromagnetic field. The porous framework increased the electromagnetic wave transmission path through repeated reflections, while the combined conductive and magnetic losses further enhanced absorption capability. Calcination temperature and FeCoNi content are critical variables; therefore, determining suitable FeCoNi dosage and calcination temperature was a major objective of this study.

Based on various characterizations and parameter analyses, the optimal microwave absorption performance is achieved at a FeCoNi doping concentration of 0.6 mol·L^−1^ and a calcination temperature of 750 °C. The test results show that at a matching thickness of 3 mm, the minimum reflection loss (RL_min_) reaches −60.89 dB, and the effective absorption bandwidth (EAB) is 6.27 GHz (7.31–13.58 GHz). When the matching thickness is 3.5 mm, the EAB can be broadened to 7.91 GHz (5.61–13.52 GHz).

The preparation process of FeCoNi/SCA developed in this study uses low-cost cassava starch as raw material, with simple and green controllable procedures, and exhibits outstanding microwave absorption performance, providing a new strategy for the development of lightweight, high-efficiency and environment-friendly microwave absorption materials. It is worth noting that this work is still in the laboratory exploration stage, and its practical application potential in industrial and military fields needs extensive verification. In future work, mechanical properties, environmental durability and other tests are required to further clarify its engineering applicability.

## Author contributions

Yue Wang: conceptualization, investigation, methodology, validation, software, data curation, formal analysis, visualization, writing – original draft. Xiang Zhou: conceptualization, investigation, methodology, validation, software, data curation, formal analysis, visualization, writing – original draft. Hu Gu: resources, test characterization. Sen Yang: esources, test characterization. Xiaoyun Long: writing – review and editing, project administration, resources, methodology, supervision, formal analysis, funding acquisition. Shuang Zhai: writing – review and editing, project administration, resources, methodology, supervision, investigation, visualization, formal analysis. Qilong Sun: writing – review and editing, methodology, formal analysis, project administration, supervision, resources, visualization, validation, investigation, funding acquisition.

## Conflicts of interest

There are no conficts to declare.

## Data Availability

The data that support the findings of this study are available from the corresponding author upon reasonable request.
